# Path planning with the derivative of heuristic angle based on the GBFS algorithm

**DOI:** 10.3389/frobt.2022.958930

**Published:** 2022-08-19

**Authors:** Daehee Lim, Jungwook Jo

**Affiliations:** R&D 2 Team, Vessel Aerospace Co., Ltd., Suwon-si, South Korea

**Keywords:** greedy best first search, heuristic angle, heuristic search, node-based algorithm, path planning, trajectory planning

## Abstract

Robots used in extreme environments need a high reactivity on their scene. For fast response, they need the ability to find the optimal path in a short time. In order to achieve this goal, this study introduces WA*DH+, an improved version of WA*DH (weighted A* with the derivative of heuristic angle). In some path planning scenes, WA*DH cannot find suboptimal nodes with the small inflation factor called the critical value due to its filtering method. It is hard to develop a new filtering method, so this study inflated the suboptimality of the initial solution instead. Critical values vary in every path planning scene, so increasing the inflation factor for the initial solution will not be the solution to our problem. Thus, WA*DH + uses the GBFS algorithm with the infinitely bounded suboptimal solution for its initial solution. Simulation results demonstrate that WA*DH + can return a better solution faster than WA*DH by finding suboptimal nodes in the given environment.

## Introduction

Path planning is an essential part of self-moving machines such as self-driving cars, unmanned aerial vehicles, or other robotics systems (De Momi, Elena et al., 2016; Boulares and Barnawi, 2021; Mac, Thi Thoa et al., 2016; Fu, Bing et al., 2018; Duan, Chao, et al., 2020; Liu, Zhiyuan et al., 2020; Hou, Mengxue et al., 2021). Path planning aims to find the path that has the lowest path cost in the shortest time because self-moving machines need fast reactions on their scene. Here, the path cost means the distance from the start to the target. Many path planning algorithms were developed to achieve this goal. Bioinspired algorithms such as genetic algorithm (GA), particle swarm optimization (PSO) (Das et al., 2016; Song et al., 2016), sampling-based algorithms such as rapidly exploring random tree (RRT), Voronoi, and artificial potential field (APF) algorithms are the examples (Yang, Liang et al., 2014; Yang, Liang et al., 2016). However, these algorithms sometimes show poor performances due to some limitations, such as the local minimum situation.

In order to avoid these threats, the A* algorithm (Hart et al., 1968) motivated by Dijkstra’s algorithm and other node-based algorithms were developed. The main characteristic of A* and all variations of A* is the heuristic. The heuristic means the estimated distance from the current node to the target. The heuristic is the most important factor in A* and all variations of A* because the heuristic can change the performance of algorithms (Jing and Yang, 2018). The concept of the heuristic is used not only for node-based algorithms but also for other algorithms, such as the ACO algorithm (Dai, Xiaolin et al., 2019).

The heuristic is a powerful method for finding the optimal path. However, algorithms using the heuristic have a time-consuming problem. This problem made A* hard to use in real-time systems, so many researchers developed various methods to get a result of heuristic-using algorithms in a short time. With these trials, weighted A*(WA*), the bounded suboptimal search algorithm, was developed (Pohl, 1970). WA* uses the heuristic multiplied by the inflation factor ( ϵ>1 ). The concept of inflating the heuristic solved a time-consuming problem. However, the inflated heuristic cannot guarantee the optimality of the result anymore.

Many researchers focused on this side effect, and as a result, many variations of WA* were developed. For example, dynamically weighted A*(DWA*) uses the *d(root)* as a depth bound (Pohl, 1973) and ${\text{A}}_{\epsilon }^{\text{*}}$ uses the desired suboptimality bound to build the focal list from a node selected to expand (Pearl and Kim, 1982; Thayer and Ruml, 2008; Thayer and Ruml, 2009). Also, Aine Sandip et al. (2016) suggested two versions of multi-heuristic A*(MHA*), which uses multiple heuristic functions to find the path; independent MHA*(IMHA*), which uses independent *g* and *h* values for each search, and shared MHA*(SMHA*), which uses different *g* but a single *g* value for all the searches. Ying et al. (2018) suggested the evolutionary heuristic A*(EHA*), which uses GA to automatically design, calibrate, and optimize multi-weighted heuristic functions to maximize the performance of the algorithm (Yiu et al, 2018).

Anytime algorithms were also used in various path planning environments. Anytime algorithms have a flexible time cost and can return a sub-optimal solution in a short time and progressively optimize it till the time limit expires. The naïve anytime A*(ATA*) returns its result by iteratively reducing the inflation factor; however, it repeats previous works (Zhou and Hansen, 2002). To address this inefficient work, anytime repairing A*(ARA*) reuses the previous work to optimize the path efficiently (Likhachev et al., 2003; Li et al., 2012). The concept of reusing the previous work was adapted to D*. Thus, anytime dynamic A* was developed (Likhachev, Maxim et al., 2005). However, they need an understanding of complex mathematical logic, which can make users reluctant to use these algorithms.

Recently, weighted A* with the derivative of the heuristic angle (WA*DH), motivated by the concept of anytime algorithms, was suggested (Lim et al., 2020). WA*DH returns its path by getting an initial solution with a certain inflation factor and partially replans the path with the same inflation factor used in the initial solution. Because WA*DH improves the initial solution only with the direction of the path, it does not require complicated mathematical logic. Thus, WA*DH has the advantage that users can easily understand how WA*DH can improve its initial solution. However, we found that the performance of WA*DH at a certain range of inflation factors is worse than that of the larger inflation factor. We supposed that this is because of the quality of the initial solution of WA*DH and this problem makes WA*DH hard to use in the self-moving vehicles used in extreme environments.

In order to address this problem, this study introduces WA*DH+. WA*DH+ is motivated by WA*DH, so the overall procedures of WA*DH+ are the same as those of WA*DH. The difference between WA*DH and WA*DH+ is that WA*DH+ uses the GBFS algorithm to get the initial solution, whereas WA*DH uses the WA* to get the initial solution. We confirmed from the simulations that the suggested method not only reduces the elapsed times but also removes the probability of the degradation of the performance of the algorithm. From the simulation results, we believe that WA*DH+ will make self-moving vehicles used in extreme environments more responsive.

## Weighted A* with the derivative of the heuristic angle

**FIGURE 1:**
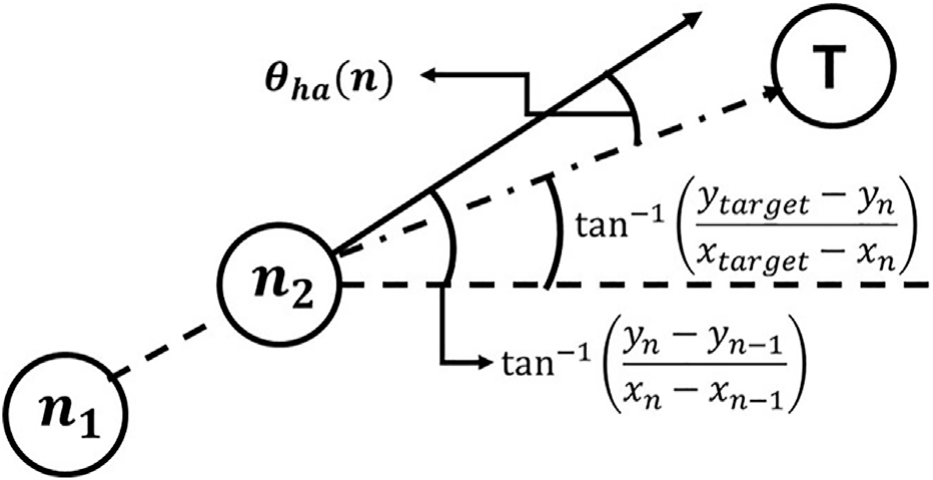
Schematic diagram for the heuristic angle.

As stated in the introduction, WA*DH uses the derivative of the heuristic angle (hereinafter referred to as DH) to refine the initial solution. The heuristic angle can be defined as (1), and its schematic diagram is stated in Figure 1. In Eq. 1, $\left\{\text{T},{\text{ n}}_{1},{\text{ n}}_{2}\right\}\in P$ denotes the target node, a current node, and the parent node of a current node, respectively. P denotes the set of nodes that consist of the path. T denotes the target, and subscripts {target, n, n−1} are subscripts of $\left\{\text{T},{\text{ n}}_{1},{\text{ n}}_{2}\right\}$ , respectively:

$$\theta_{\mathrm{ha}}=\left|\mathrm{ta}n^{-1}\left(\frac{y_{\mathrm{target}}-y_{n}}{x_{\mathrm{target}}-x_{n}}\right)-\mathrm{ta}n^{-1}\left(\frac{y_{n}-y_{n-1}}{x_{n}-x_{n-1}}\right)\right|$$ (1)

**FIGURE 2:**
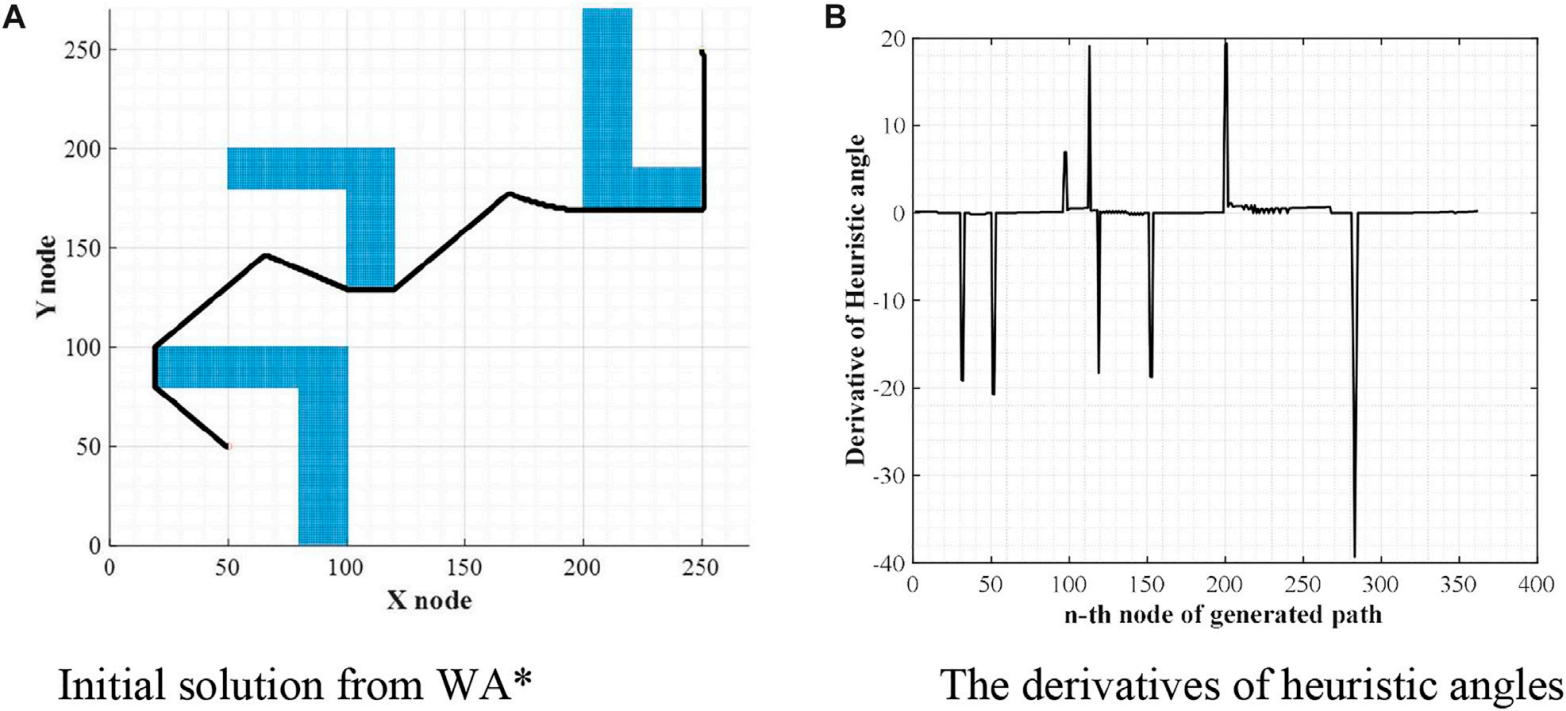
Initial solution and their derivatives of heuristic angles. **(A)** Initial solution from WA*. **(B)** The derivatives of heuristic angles.

The first step of WA*DH is getting an initial solution from WA*. Once the procedures of WA* are executed, coordinates of nodes that consist of the initial solution will be listed. With this list, WA*DH calculates heuristic angles and their derivatives. The shape of the initial solution and the derivatives of heuristic angles are stated in Figures 2A,B.

**FIGURE 3:**
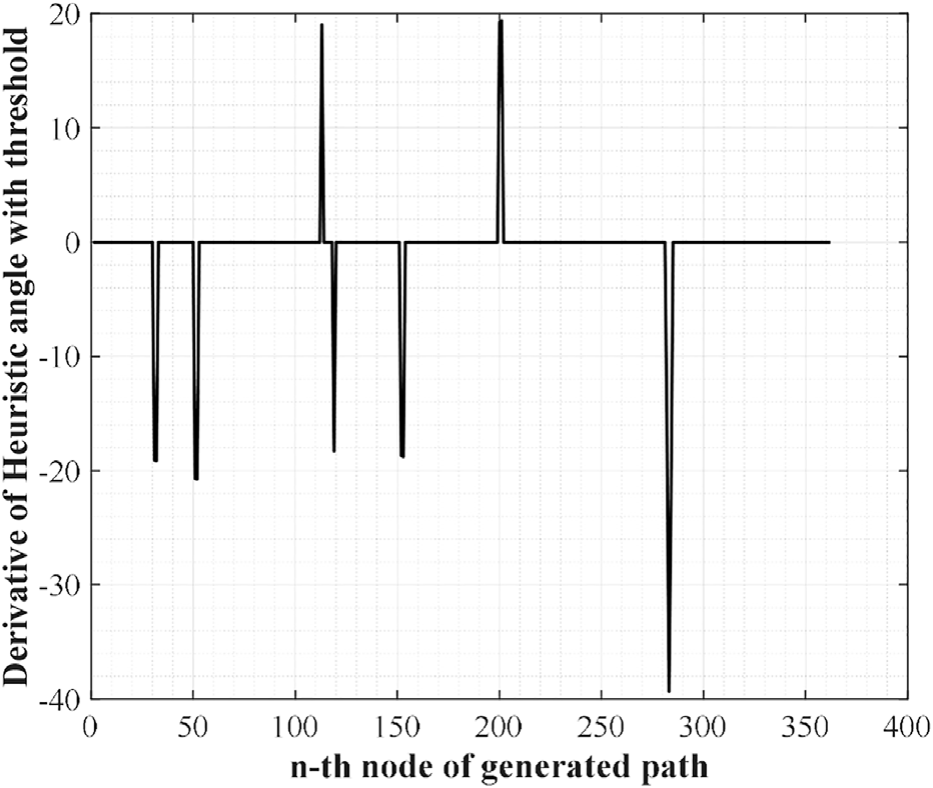
Filtered DH with a threshold.

From Figure 2B, there are some noise-shaped patterns near 0. These elements can be considered suboptimal nodes by the definition of suboptimal nodes, so they must be eliminated. To do so, WA*DH filters them with a threshold defined as (2). With these methods, Figure 2B will be changed to Figure 3. In Eq. 2, ${\theta^{\prime }}_{ha}\left(n\right)$ denotes the derivative of the heuristic angle of $n_{th}$ node of the initial solution and *m* denotes the total number of nodes that consist of the initial solution:

$$Threshold=\sum_{n=1}^{m}\left({\theta^{\prime }}_{ha}\left(n\right)\right)÷m\;$$ (2)

The next step of WA*DH is searching suboptimal nodes. The suboptimal node contains two nodes: occurrence and escape. The occurrence node is defined as a node with a positive DH, and the escape node is defined as a node with negative DH. Also, the escape node must satisfy one more condition; there must be at least one occurrence node between the start and a node with negative DH. By the definition of suboptimal nodes, we can find two occurrence nodes near the 110th and 200th nodes and three escape nodes near the 120th, 150^th^, and 280th nodes from Figure 3.

**FIGURE 4:**
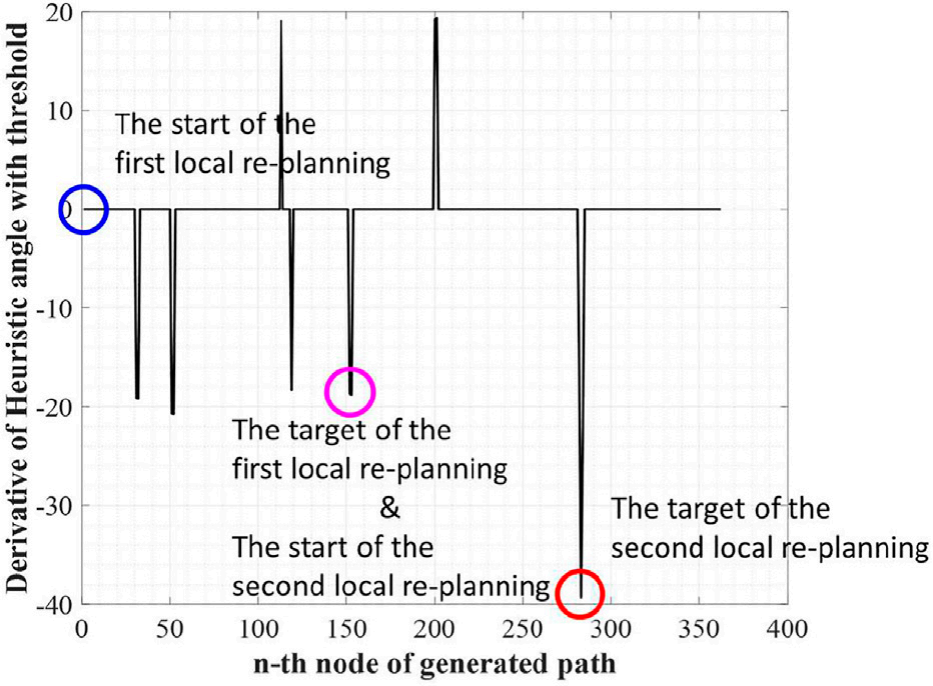
The start and the target for local replanning of the example.

The purpose of searching suboptimal nodes is to make the node-set. The node-set contains the start and the target nodes for local replanning. Local replanning needs to be executed for the number of occurrence nodes. In the example environment, there are two occurrence nodes, so local replanning needs to be executed twice. The start and the target for local replanning can be determined as follows and Figure 4 shows the start and the target for local replanning of the example environment:

1) The start of the first local replanning is the start of the initial solution, and the target of the first local replanning is the first escape node. However, if there are two or more escape nodes between two occurrence nodes (or target of the initial solution), the last escape node will be chosen for the target of the local replanning.
2) The start and the target of the second and the subsequence local replanning are targets of the previous local replanning and $n_{th}$ escape node. If there are two or more escape nodes between two occurrence nodes (or target of the initial solution), the last escape node will be chosen for the target of the local replanning.

**FIGURE 5:**
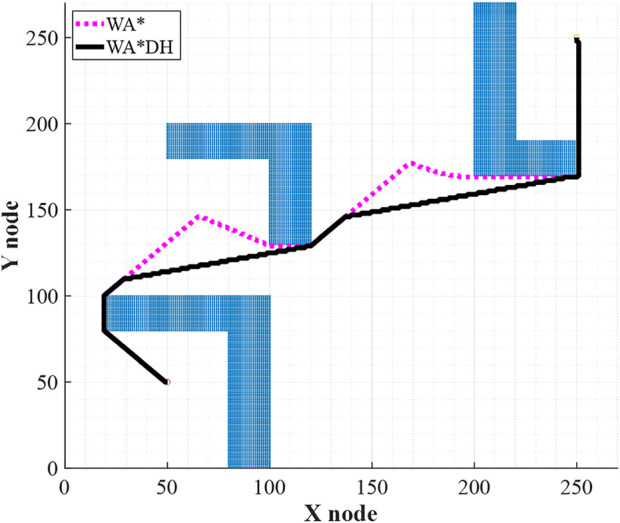
Initial and final solution of WA*DH.

Next, WA*DH executes WA* multiple times based on the node-set. The number of executions is the same as the number of occurrence nodes, and the inflation factor in each execution is equal to the initial solution. After that, WA*DH replaces the initial solution with the locally replanned paths. The procedure of the replacement contains not only replacing nodes but also updating *g(n)* and *h(n)*. As a result, WA*DH returns its result. Figure 5 states the initial solution and final solution of WA*DH in a dotted line and a full line, respectively.

## WA*DH+: Locally replans paths based on the infinitely bounded suboptimal solution

### Critical values on WA*DH

**FIGURE 6:**
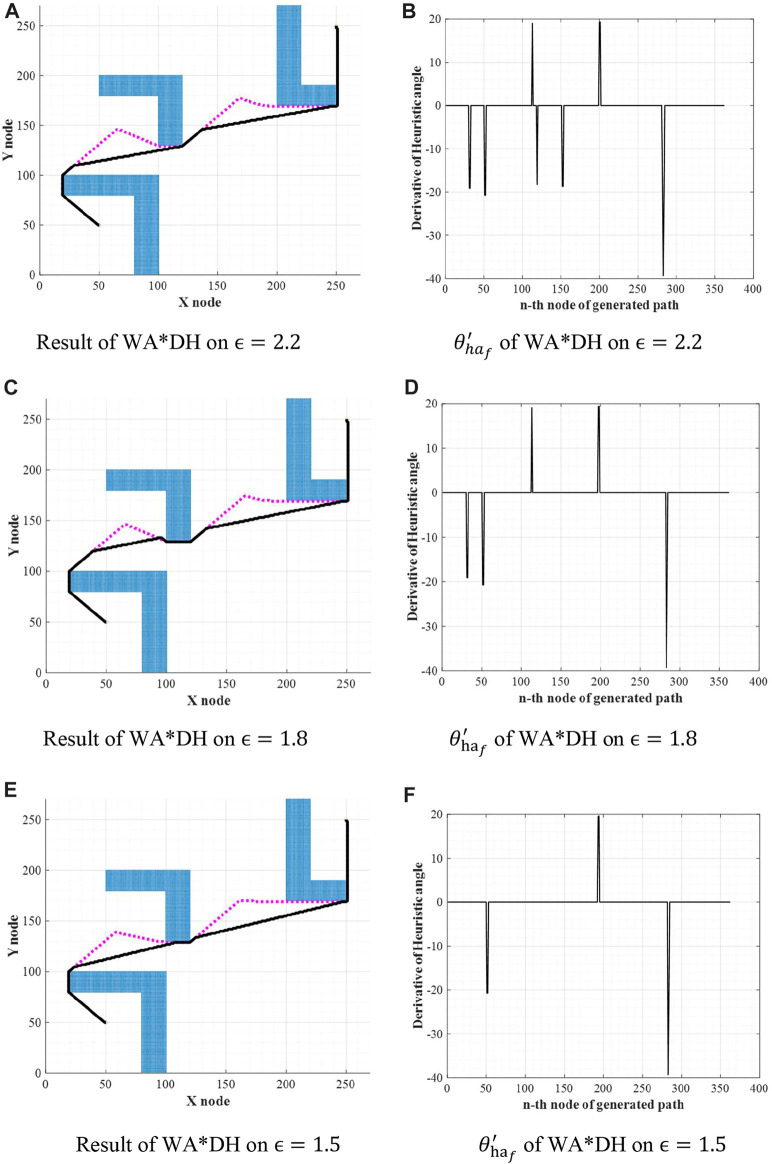
Results of WA*DH on the different inflation factors. **(A)** Result of WA*DH on ϵ=2.2 . **(B)** ${\theta^{\prime }}_{ha_{f}}$ of WA*DH on ϵ=2.2 . **(C)** Result of WA*DH on ϵ=1.8 . **(D)** ${\theta^{\prime }}_{{\text{ha}}_{f}}$ of WA*DH on ϵ=1.8 . **(E)** Result of WA*DH on ϵ=1.5 . **(F)** ${\theta^{\prime }}_{{\text{ha}}_{f}}$ of WA*DH on ϵ=1.5 .

Theoretically, the path cost of WA*DH decreases with the decreasing inflation factor. However, we found that the path cost of WA*DH with a certain inflation factor is higher than that with a larger inflation factor. This is stated in Figure 6 and Table 1. In Figures 6B,D,F, ${\theta^{\prime }}_{ha\;f}$ denotes the filtered DH with a threshold; a dotted line and a full line in Figures 6A,C,E denote the initial and final solution of WA*DH, respectively. Table 1 states the elapsed times and path costs of each simulation.

**TABLE 1:** Elapsed times and costs Figure 4.

Inflation factor	Time (s)	Cost (node)
2.2	2.04	404.84
1.8	2.49	408.15
1.5	4.08	404.84
1 (= ${\text{A}}^{\text{*}}$ )	15.40	404.84

From the initial solution in Figures 6A,C,E, it is intuitively clear that there are two detouring sections, so we can expect that there will be two escape nodes. When ϵ=2 , WA*DH detected two escape nodes correctly, so the quality of the path cost is equal to A*. However, when ϵ=1.8 , WA*DH detected only one escape node near the $300_{th}$ node and the path cost of WA*DH is higher than the path cost of WA*DH when ϵ=2 . We first supposed this is because WA*DH cannot detect all suboptimal nodes. However, as stated in Figure 6D, WA*DH with ϵ=1.5 found only one escape node at the same location in Figure 6D, but its path cost is equal to A*. From these results, we concluded that the fault detection of suboptimal nodes is not the cause of the poor performance. Also, we defined inflation factors that make performance degradation critical value.

### Get an initial solution from the greedy GFS algorithm

It is hard to avoid the threat of critical values because they are unpredictable. We thought that using a large inflation factor would avoid the threat of the critical value. However, it is hard to decide on a large inflation factor because the criteria for big and small are different for each person. From this, we hypothesized that an extremely high inflation factor, such as an infinite inflation factor, will be enough to call a large inflation factor. Therefore, we suggest the greedy best-first search (GBFS) algorithm as the algorithm for the initial solution. GBFS is an algorithm that searches nodes with only heuristic, so we thought that using the GBFS algorithm is equal to using WA* with the infinite inflation factor. The cost function of GBFS is stated in Eq. 3, where *f(n)* denotes the cost function of a node of the GBFS algorithm and *h(n)* denotes the heuristic of a node:

f(n)=h(n) (3)

*Theorem*. If the inflation factor is extremely high (or infinite), then the effect of the *g* term will be disappeared. Here, *g* is the cost of the path from the start node to the $n_{th}$ node.

*Proof*.

Let the cost function of an algorithm be

f(ϵ)=g+ϵ×h (4)

We can change Eq. 4 to

$$\frac{f\left(\epsilon \right)}{\epsilon }=\frac{g}{\epsilon }+h$$ (5)

Taking the limit on both sides of Eq. 5,

$$\underset{\epsilon \to \infty }{\lim}\left(\frac{f(\epsilon )}{\epsilon }\right)=\;\underset{\epsilon \to \infty }{\lim}\left(\frac{g}{\epsilon }\right)+\;\underset{\epsilon \to \infty }{\lim}\left(h\right)$$ (6)

$$\frac{f\left(\epsilon \right)}{\epsilon }\approx h$$ (7)

∴f(ϵ)≈ϵ×h (8)

The role of the inflation factor on the cost function, such as Eq. 4, is deciding the influence of the heuristics compared to the cost of the path, *g(n)*. However, there is no need for the inflation factor because Eq. 8 does not contain *g(n)* anymore. so we can remove ϵ from Eq. 8 With these procedures, we can derive Eq. 3 as a result.

**FIGURE 7:**
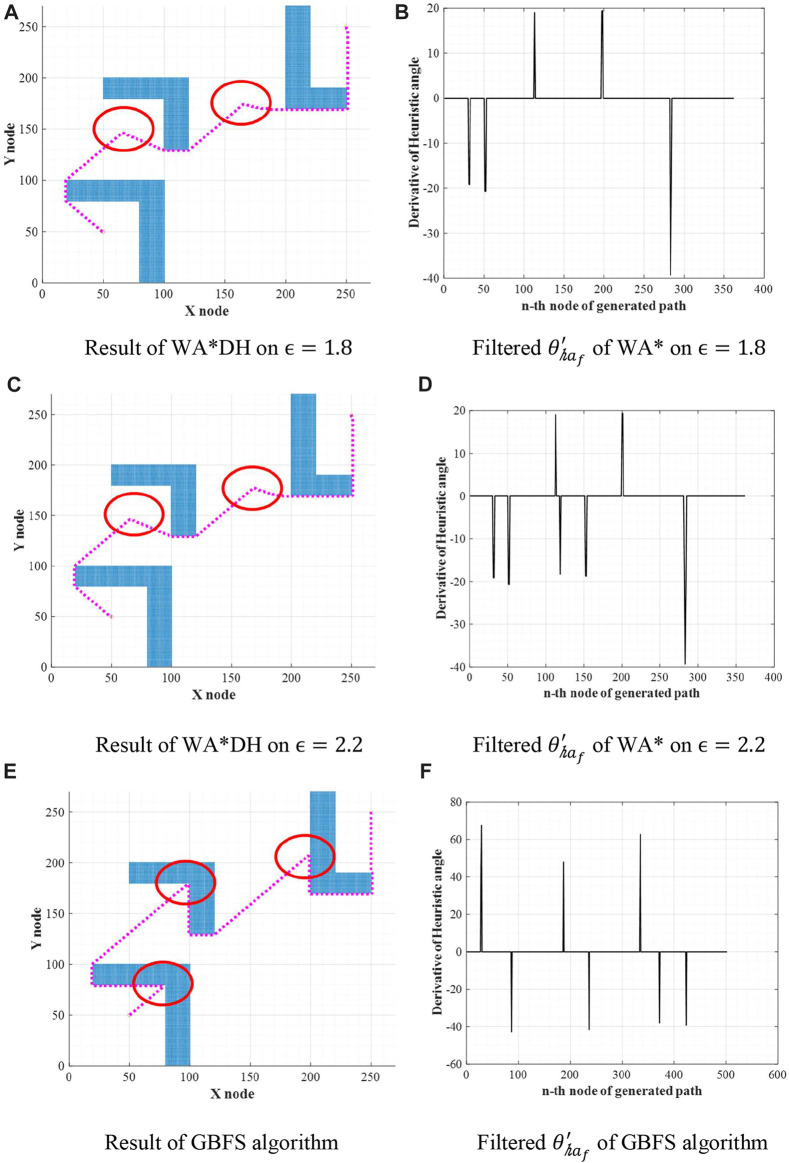
Escape nodes of WA* on different inflation factors. **(A)** Result of WA*DH on ϵ=1.8 . **(B)** Filtered ${\theta^{\prime }}_{ha_{f}}$ of WA* on ϵ=1.8 . **(C)** Result of WA*DH on ϵ=2.2 . **(D)** Filtered ${\theta^{\prime }}_{ha_{f}}$ of WA* on ϵ=2.2 . **(E)** Result of the GBFS algorithm. **(F)** Filtered ${\theta^{\prime }}_{ha_{f}}$ of the GBFS algorithm.

Using the GBFS algorithm gives us some advantages, as stated in Figure 7. Figure 7 states escape nodes found from the result of WA* with ϵ= 1.8, ϵ=2.2, and the GBFS algorithm, and circles in Figures 7A,C,E state the locations of occurrence nodes.

WA*DH in Figure 7B detected two occurrence nodes, so we can expect there would be two escape nodes. However, WA*DH in Figure 7 detected only one escape node. Also, in Figure 7D, WA*DH detected two occurrence nodes and two escape nodes. Moreover, WA*DH in Figure 7F found three occurrence nodes and escape nodes. In fact, considering the placement of obstacles in the simulation environment of Figure 7, there must be three occurrence nodes and escape nodes. However, Figures 7B,D could not detect all suboptimal nodes due to the relatively high optimality of WA*. From these results, we can prove that the high inflation factor can detect suboptimal nodes clearly.

The path planning with the GBFS algorithm is also very useful in terms of elapsed time. The elapsed time of WA* gets shorter as the inflation factor increases. This means that an infinite inflation factor can theoretically get a result of WA* in the fastest time. Thus, we can expect that the GBFS algorithm can reduce the elapsed time of our algorithm, WA*DH+. This will be simulated in Section 4.

### Procedures of WA*DH+

This section introduces our algorithm, WA*DH+, and how WA*DH + gets its result. Procedures of WA*DH + are similar to those of WA*DH: getting an initial solution, finding escape nodes, locally replanning the paths, and connecting them. The details of procedures of WA*DH + are stated below.

First of all, WA*DH + gets an initial solution from the GBFS algorithm. Unlike WA*DH, WA*DH + does not need to decide the inflation factor for the initial solution. After getting an initial solution, then WA*DH+ calculates ${\theta^{\prime }}_{ha\;f}$ with the moving median filter and the threshold.

The next step of WA*DH+ is finding the suboptimal nodes to decide the start and the targets for local replanning. Next, WA*DH+ executes the local replanning. In this procedure, WA*DH+ needs to decide on an inflation factor.

**FIGURE 8:**
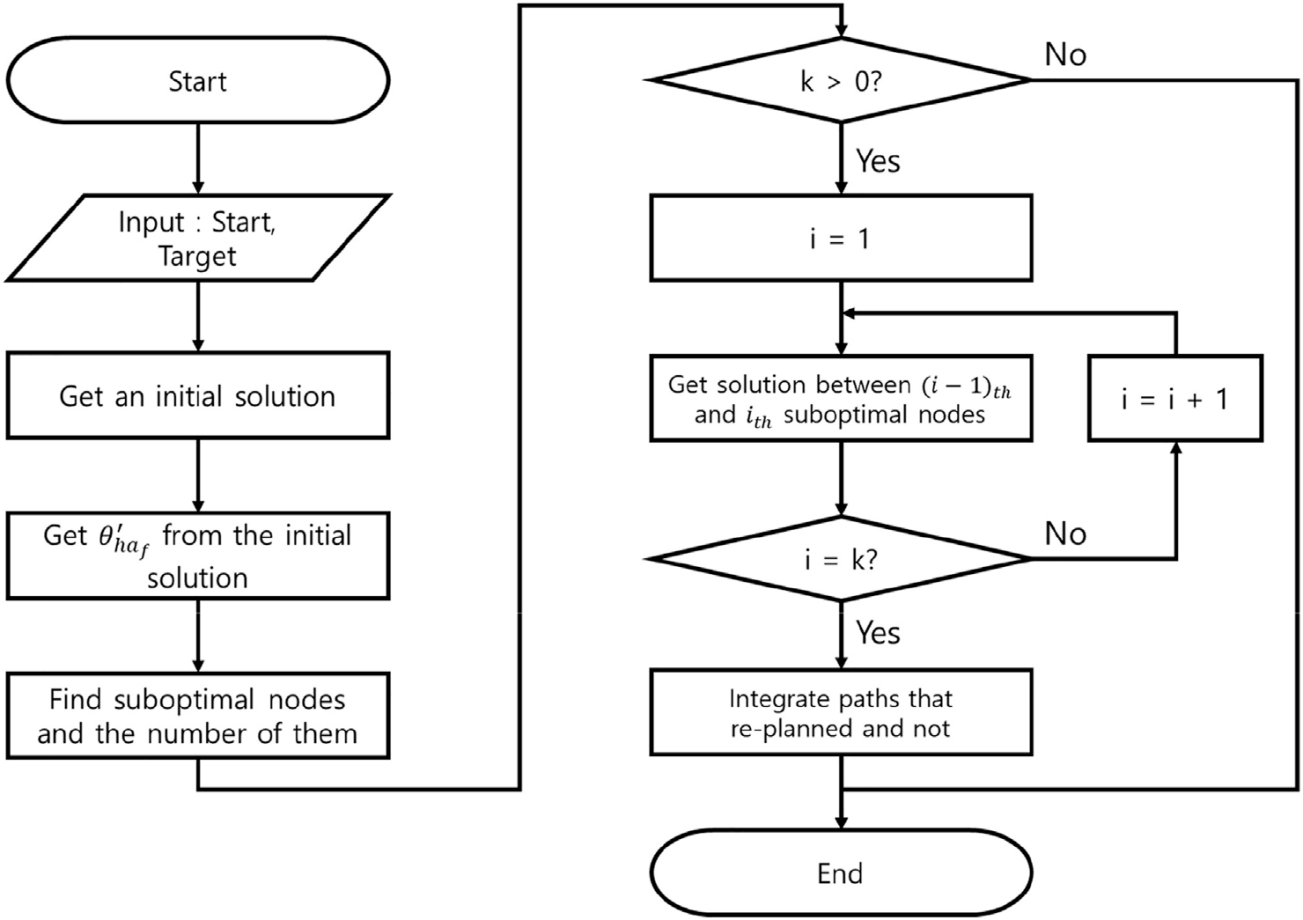
Flowchart of WA*DH+.

**FIGURE 9:**
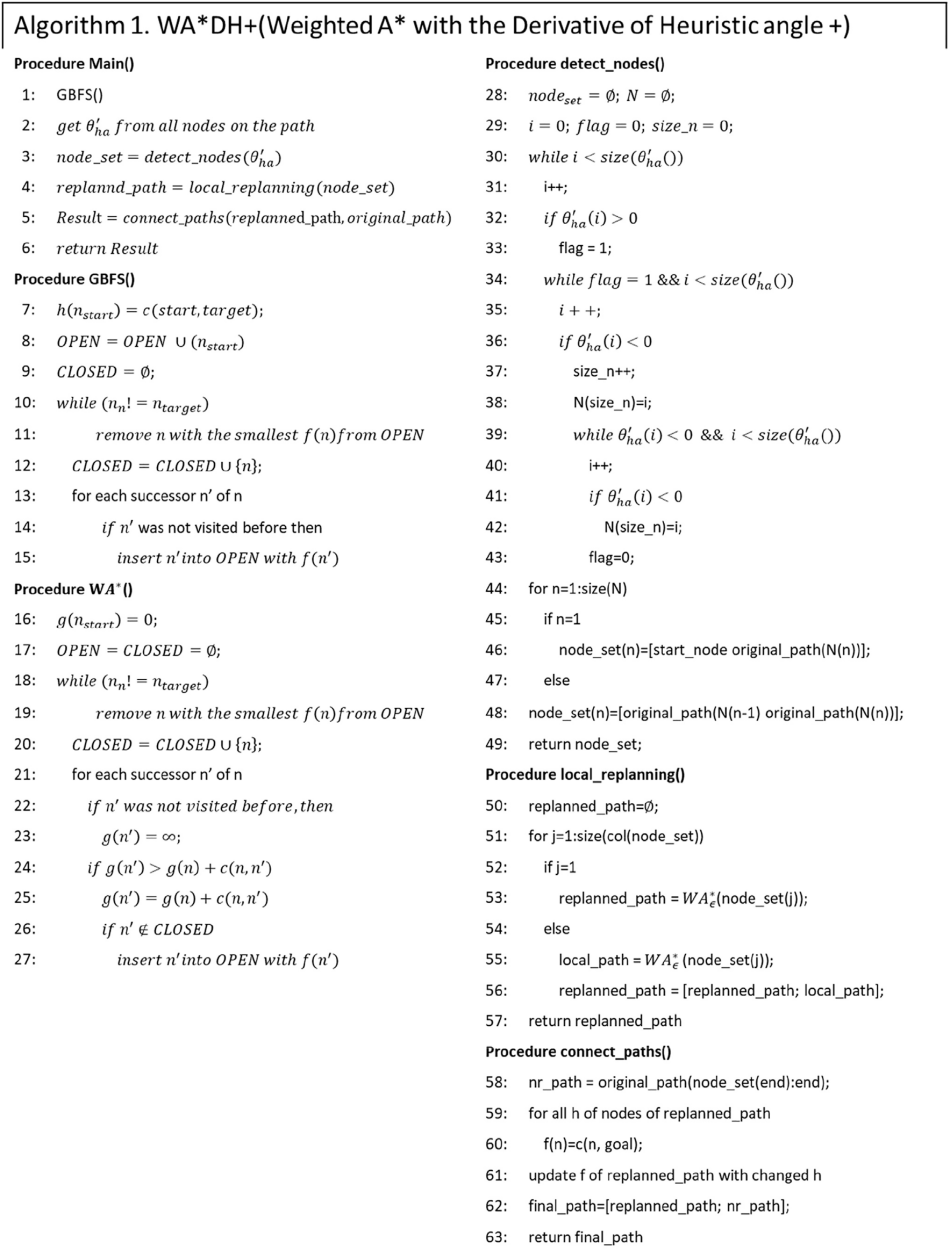
Pseudocode of WA*DH+.

The final step of WA*DH+ is path-connecting. In this procedure, WA*DH + needs to update only heuristics, whereas WA*DH needs to update the actual costs, *g(n)*, and heuristics. Also, WA*DH+ needs an additional procedure to calculate the path cost, whereas WA*DH can get its path cost from *g(n)* of the target. After calculating the path cost, WA*DH+ can finally get its result. The pseudocode and the flowchart of WA*DH+ are stated in Figures 8, 9. In the flowchart, when  i=1 , the ${\left(i-1\right)}_{th}$ node is equal to the original start node.

## Simulation results

### Simulation environments

**FIGURE 10:**
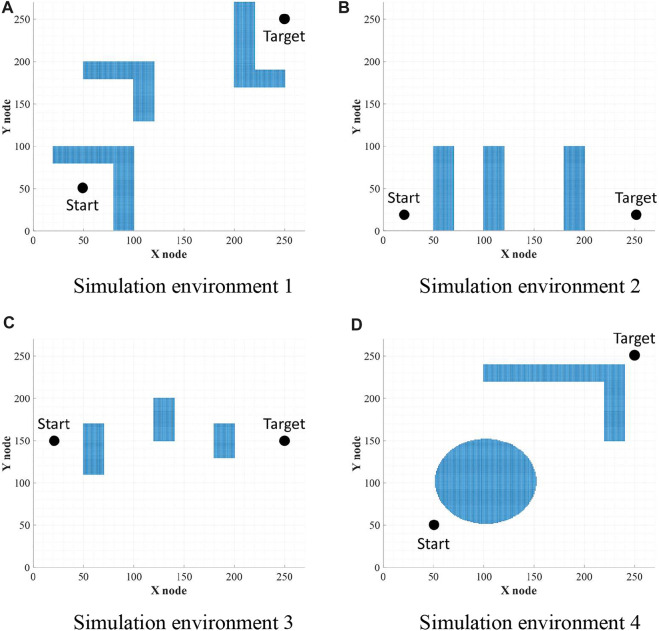
Environments for simulations. **(A)** Simulation environment 1. **(B)** Simulation environment 2. **(C)** Simulation environment 3. **(D)** Simulation environment 4.

In order to compare the performances with WA*DH, obstacles were placed the same as in the simulation environments of WA*DH, and the sizes of all simulation environments are also the same as those of the simulation environments of WA*DH (270 x 27 nodes) as stated in Figure 10. All simulations were conducted in MATLAB with Windows 10, i7-9700 CPU with 32 GB RAM, and there are no acceleration methods or parallel processes such as Graphics Processing Unit (GPU) and parallel processing with CPU cores.

### Performances of WA*DH+

**FIGURE 11:**
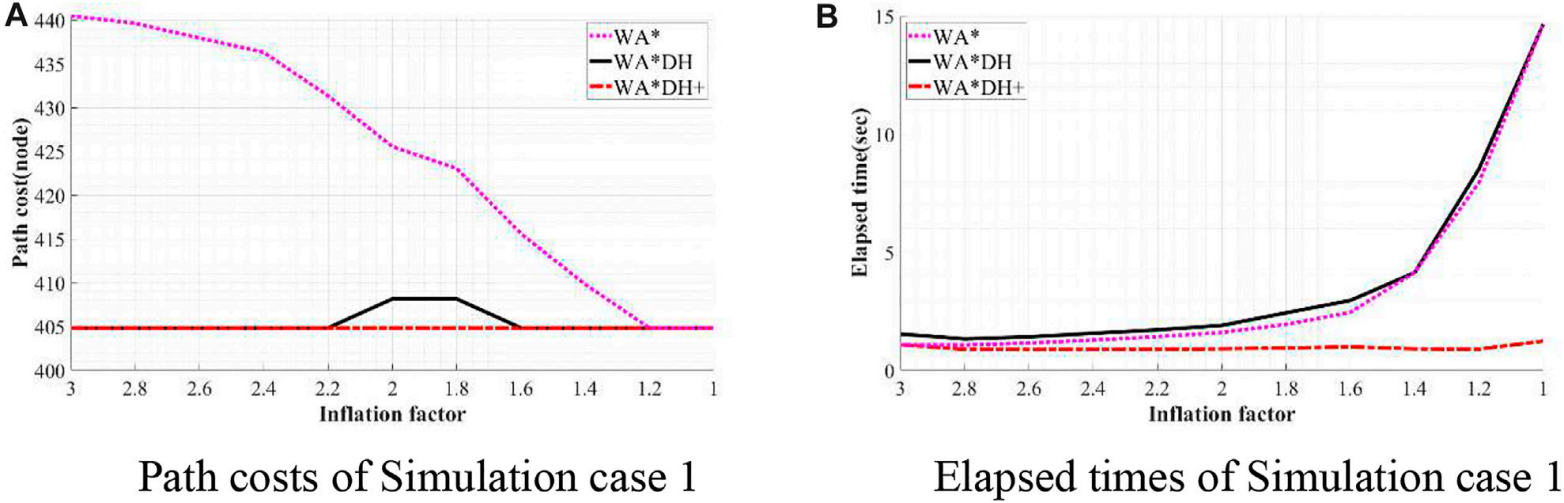
Result of simulation case 1. **(A)** Path costs of simulation case 1. **(B)** Elapsed times of simulation case 1.

**FIGURE 12:**
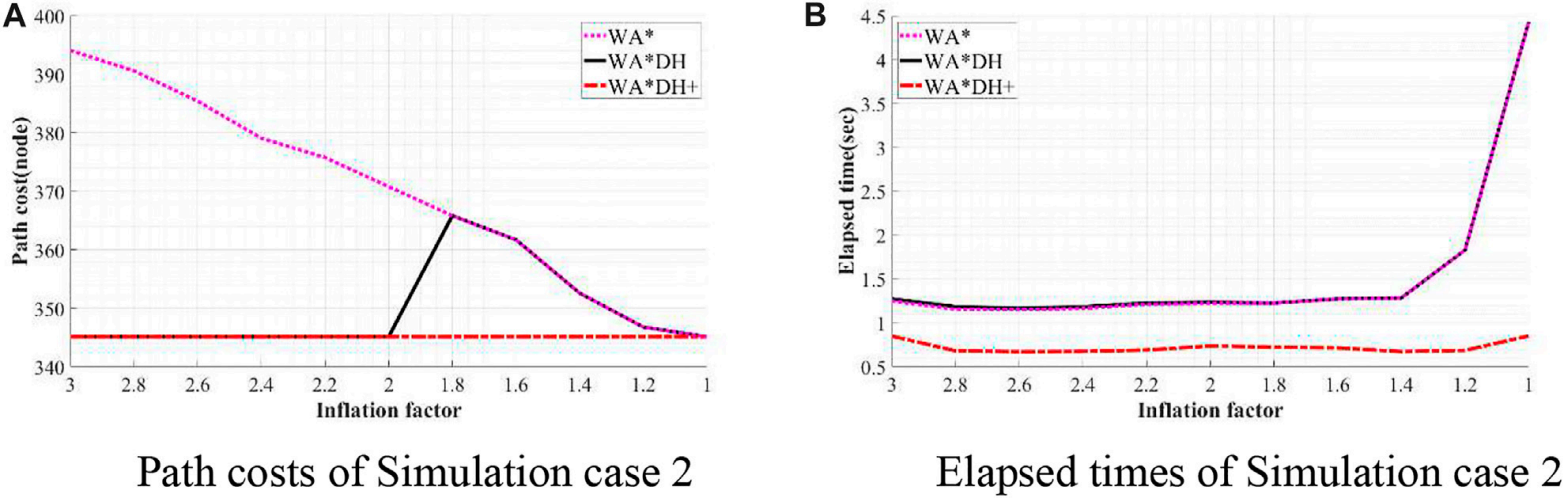
Result of simulation case 2. **(A)** Path costs of simulation case 2. **(B)** Elapsed times of simulation case 2.

**FIGURE 13:**
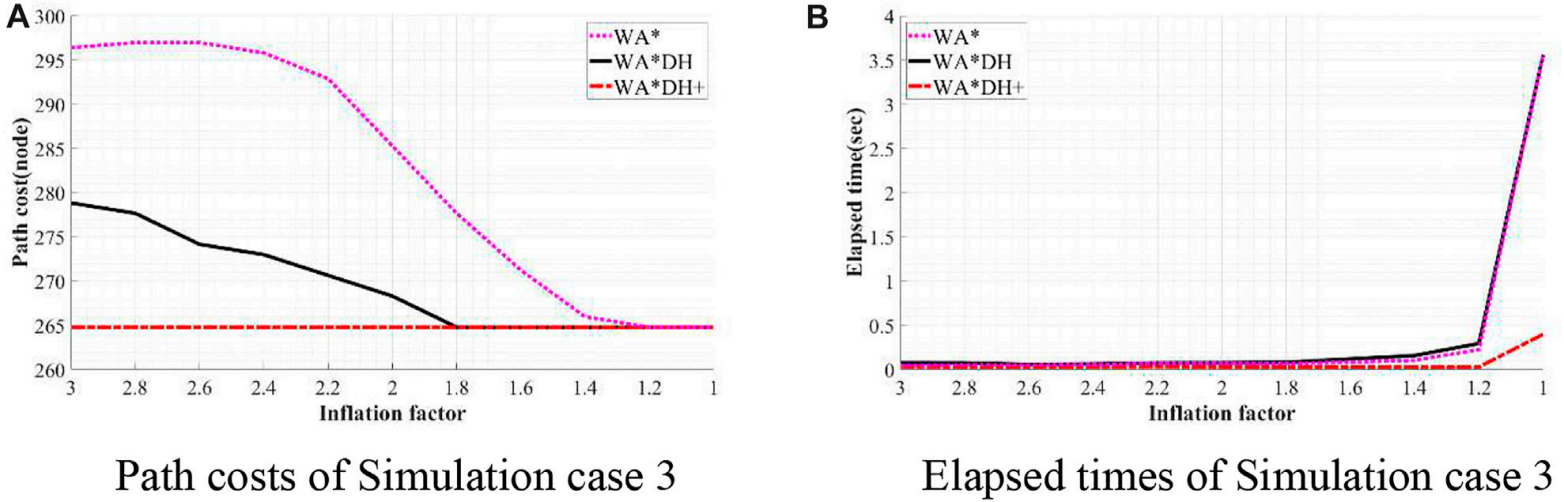
Results of simulation case 3. **(A)** Path costs of simulation case 3. **(B)** Elapsed times of simulation case 3.

**FIGURE 14:**
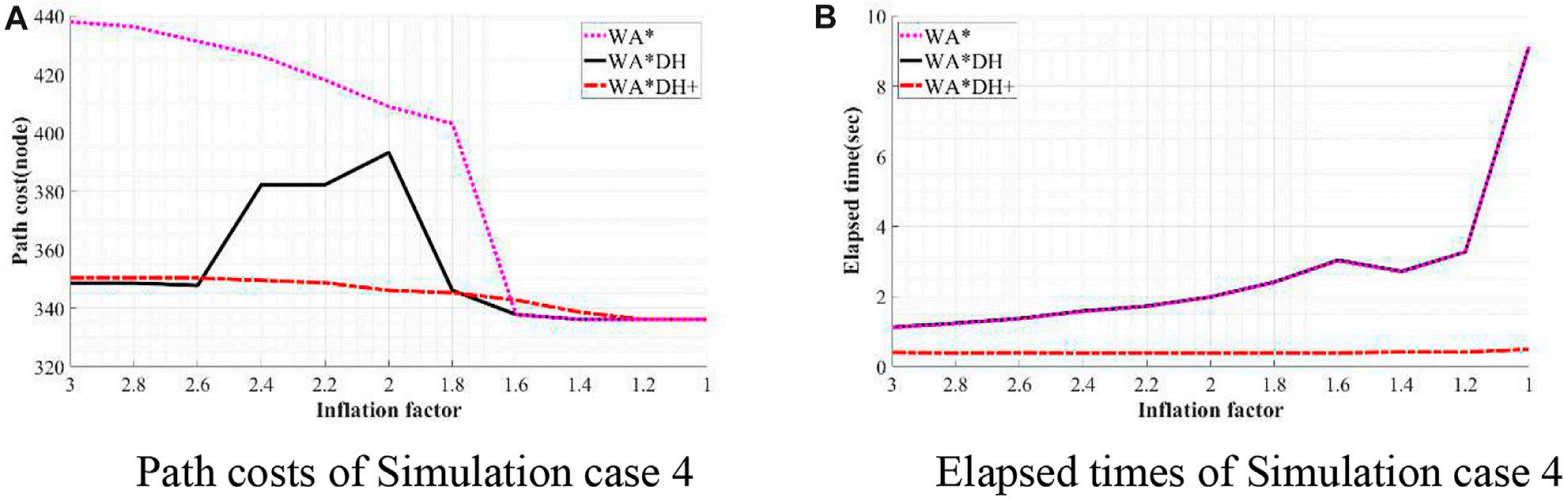
Result of simulation case 4. **(A)** Path costs of simulation case 4. **(B)** Elapsed times of simulation case 4.

To validate the performance of WA*DH+, we simulated WA*DH+, WA*DH, and WA* in terms of the path cost and elapsed time in each environment. Simulations were conducted by reducing the inflation factor from 3 to 1 by 0.2 to compare performances near critical values. Results of simulations are stated in Figures 11–14, and quantitative comparisons are stated in Tables 2–5.

**TABLE 2:** Quantitative comparison of the performances of algorithms on simulation 1.

€	WA*DH+		WA*DH		WA*	
	Cost (node)	Time (s)	Cost (node)	Time (s)	Cost (node)	Time (s)
3	404.84	1.08	404.84	1.54	440.46	1.08
2.8	404.84	0.88	404.84	1.33	439.63	1.07
2.6	404.84	0.88	404.84	1.41	437.97	1.15
2.4	404.84	0.88	404.84	1.57	436.32	1.28
2.2	404.84	0.88	404.84	1.71	431.35	1.42
2.0	404.84	0.91	408.15	1.90	425.55	1.60
1.8	404.84	0.94	408.15	2.43	423.06	1.94
1.6	404.84	0.99	404.84	2.96	415.61	2.46
1.4	404.84	0.90	404.84	4.13	409.81	4.14
1.2	404.84	0.89	404.84	8.52	404.84	7.95
1.0	404.84	1.24	404.84	14.65	404.84	14.65

From Table 2, the path cost of WA*DH with a relatively large inflation factor (> 2.2) is the same path cost of A*’s. However, when ϵ is in the range from 2.2 to 1.6, the path cost of WA*DH is about 0.81% larger than that with larger inflation factors. In contrast, the path cost of WA*DH + does not change with varying inflation factors, and its quality keeps the same as the path cost of A*.

Table 3 shows that path costs of WA*DH and WA*DH+ with relatively high inflation factors (> 2.0) are the same as the path cost of A*. However, when the inflation factor is lower than 2, the path cost of WA*DH is the same as WA* until the inflation factor decreases to 1, whereas the path cost of WA*DH + does not change with varying inflation factors.

**TABLE 3:** Quantitative comparison of the performances of algorithms on simulation 2.

€	WA*DH+		WA*DH		WA*	
	Cost (node)	Time (s)	Cost (node)	Time (s)	Cost (node)	Time (s)
3	345.14	0.85	345.14	1.27	394.13	1.25
2.8	345.14	0.68	345.14	1.18	390.62	1.15
2.6	345.14	0.67	345.14	1.16	385.44	1.15
2.4	345.14	0.68	345.14	1.18	379.1	1.17
2.2	345.14	0.69	345.14	1.22	375.79	1.21
2.0	345.14	0.74	345.14	1.23	370.82	1.22
1.8	345.14	0.72	365.85	1.23	365.85	1.23
1.6	345.14	0.72	361.71	1.27	361.71	1.27
1.4	345.14	0.67	352.59	1.29	352.59	1.29
1.2	345.14	0.69	346.79	1.83	346.79	1.83
1.0	345.14	0.85	345.14	4.43	345.14	4.43

**TABLE 4:** Quantitative comparison of the performances of algorithms on simulation 3.

€	WA*DH+		WA*DH		WA*	
	Cost (node)	Time (s)	Cost (node)	Time (s)	Cost (node)	Time (s)
3	264.79	0.03	278.85	0.08	296.43	0.05
2.8	264.79	0.03	277.68	0.08	297.01	0.05
2.6	264.79	0.03	274.17	0.06	297.01	0.05
2.4	264.79	0.03	273.00	0.06	295.84	0.06
2.2	264.79	0.04	270.65	0.08	292.91	0.07
2.0	264.79	0.03	268.31	0.08	285.3	0.07
1.8	264.79	0.03	264.79	0.08	277.68	0.07
1.6	264.79	0.03	264.79	0.12	271.24	0.08
1.4	264.79	0.03	264.79	0.16	265.97	0.10
1.2	264.79	0.03	264.79	0.29	264.79	0.23
1.0	264.79	0.40	264.79	3.56	264.79	3.56

**TABLE 5:** Quantitative comparison of the performances of algorithms on simulation 4.

€	WA*DH+		WA*DH		WA*	
	Cost (node)	Time (s)	Cost (node)	Time (s)	Cost (node)	Time (s)
3	350.33	0.40	348.58	1.12	437.97	1.11
2.8	350.33	0.39	348.58	1.23	436.32	1.23
2.6	350.33	0.40	347.75	1.37	431.34	1.36
2.4	349.50	0.38	382.23	1.59	426.37	1.57
2.2	348.68	0.39	382.23	1.73	418.09	1.71
2.0	346.09	0.39	393.24	1.99	409.00	1.98
1.8	345.26	0.39	346.09	2.41	403.18	2.40
1.6	342.78	0.39	337.81	3.03	337.81	3.02
1.4	338.63	0.41	336.15	2.72	336.15	2.69
1.2	336.15	0.41	336.15	3.27	336.15	3.27
1.0	336.15	0.49	336.15	9.13	336.15	9.13

In the case of simulation case 3, the path cost of WA*DH+ is about 5.04% lower than that of WA*DH. Thus, we can see that WA*DH + not only removes the risk of a critical value but also returns a lower path cost at a relatively high inflation factor. Moreover, we also confirmed that the path cost of WA*DH + keeps the same quality as the path cost of A* regardless of the inflation factor.

Unlike other results of simulation cases, when ϵ=3 in case of simulation case 4, the path cost of WA*DH+ is about 0.5% higher than that of WA*DH, and this difference rises to about 1.47% when ϵ=1.6 . However, it is hard to say that the quality of WA*DH+ is bad because the purpose of using WA*DH+ is to avoid the threat of the critical value.

Besides being free from the threat of the critical value, we also found that WA*DH + has an advantage in terms of the elapsed time. In all simulation cases, elapsed times of WA*DH increase exponentially near ϵ=1 . However, the elapsed time of WA*DH + does not significantly increase with decreasing inflation factor, and elapsed times of WA*DH + are much lower than WA*DH. In fact, considering that using the GBFS algorithm has the same meaning as using the infinite inflation factor at WA*, it is a natural result because the higher the inflation factor is, the faster the result can be returned.

## Discussion

This study aims to evade the threat of the critical values on WA*DH. We found that the critical value comes from the fault detection of suboptimal nodes from the initial solution with relatively high optimality. We hypothesized that high suboptimality could find suboptimal nodes clearly, so we suggested our algorithm, WA*DH +, which uses the GBFS algorithm for the initial solution. From simulations, it can be proven that WA*DH + can avoid the threat of the critical value successfully by detecting suboptimal nodes more clearly than WA*DH. In terms of the elapsed time, we also confirmed that the elapsed time does not change significantly with varying inflation factors; however, the elapsed time of WA*DH increases exponentially when the inflation factor is near 1.

Although WA*DH + shows powerful performances in terms of the path cost and the elapsed time, WA*DH + still cannot guarantee admissibility because WA*DH + refines the initial solution based on the GBFS algorithm that has the infinitely bounded suboptimality. It will remain a limitation of algorithms using the concept of WA*DH. Also, WA*DH+ cannot refine the initial solution if there are circular obstacles in the path planning scene. However, we expect this will be removed in future works using new filtering methods of DH or additional procedures to the result of WA*DH+.

## Data Availability

The original contributions presented in the study are included in the article. Further inquiries can be directed to the corresponding author.
